# Supplementary material for: Maternal and Infant Health in Abu Dhabi: Insights from Key Informant Interviews
Source: Int J Environ Res Public Health. 2019 Aug 22;16(17):3053. doi: 10.3390/ijerph16173053 (PMC6747237; doi:10.3390/ijerph16173053)
Supplement: Supplementary file 1 [file ijerph-16-03053-s001.pdf]

Table S1. The steps in method from Burnard [1] for analysing interview transcripts in qualitative research.

Stage	Activity
1	Notes were made during and after each interview of the interviewer's impressions relating to the main topics and themes, as well as any additional factors about which the interviewer was aware.
2	Transcripts were carefully read and notes were made on emergent themes and key issues. This step sought to immerse the researcher in the data as it was important that the researcher was aware of the participants' perspectives on Emirati maternal and infant health.
3	Open coding undertaken to identify categories or headings which covered all or most of the data in the interview transcripts.
4	Reduction of the number of categories by identifying broader headings and including sub-headings.
5	A final list of categories without overlap or repetition was produced.
6	Validation of the categorisation by comparing lists made by two colleagues who read the transcripts and compiled category lists.
7	The transcripts were again checked to ensure an exhaustive list of categories had been identified.
8	Transcripts coded.
9	Transcripts checked to ensure that the participants' responses remained within context.
10	Coded sections of transcripts organised into categories and sub-headings.
11	Some of the respondents were then asked if they agreed with the coding of their statements to re-check the validity of the categorisation process.
12	Writing up results: all information available.
13	Writing up: accuracy.
14	Relating the results of the interviews to the results reported from the original study.

1. Burnard, P., A method of analysing interview transcripts in qualitative research. *Nurse Educ. Today* **1991,** 11, (6), 461-466.
